# Negative association between Body Roundness Index and bone mineral density: insights from NHANES

**DOI:** 10.3389/fnut.2024.1448938

**Published:** 2024-08-08

**Authors:** Ziyao Ding, Zhe Zhuang, Rongze Tang, Xinzhe Qu, Zicheng Huang, Maji Sun, Feng Yuan

**Affiliations:** ^1^Department of Spine Surgery, Affiliated Hospital of Xuzhou Medical University, Xuzhou, Jiangsu, China; ^2^First Clinical Medical College, Xuzhou Medical University, Xuzhou, Jiangsu, China; ^3^Department of Gynecologic Oncology, International Peace Maternity & Child Health Hospital, School of Medicine, Shanghai Jiao Tong University, Shanghai, China

**Keywords:** Body Roundness Index (BRI), bone mineral density (BMD), osteoporosis, NHANES, obesity

## Abstract

**Background:**

Osteoporosis (OP), affecting millions around the globe, is a prevalent degenerative condition of the bones characterized by a decrease in bone mineral density (BMD) and an increase in bone fragility. A novel anthropometric measure, the Body Roundness Index (BRI), provides a more accurate assessment of body fat distribution compared to traditional metrics. Using data from the National Health and Nutrition Examination Survey (NHANES), this study aims to explore the relationship between BRI and total BMD in U.S. adults aged 20 and above.

**Methods:**

Data from NHANES (2011–2018) were examined, encompassing 9,295 participants following exclusions. Dual-energy X-ray absorptiometry (DXA) was employed to measure BMD. BRI was calculated using waist circumference (WC) and height. The study accounted for variables such as demographic traits, physical exam results, lab test findings, and survey responses. Weighted multivariable linear regression models and smooth curve fitting methods were utilized to assess the relationship between BRI and total BMD.

**Results:**

The research found a notable inverse relationship between BRI and total BMD. In the model with full adjustments, an increase of one unit in BRI was linked to a 0.0313 g/cm^2^ reduction in total BMD (*P* < 0.0001). Moreover, an inflection point was identified at BRI = 9.5229, where each one-unit rise in BRI beyond this threshold corresponded to a more substantial decrease in total BMD (0.0363 g/cm^2^). Analysis by subgroups revealed that this negative association was consistent across most demographic and health-related categories.

**Conclusions:**

The results demonstrate a notable inverse relationship between BRI and total BMD, indicating that a higher BRI could be associated with lower BMD and a potentially greater risk of developing OP. This underscores the significance of accounting for body fat distribution in preventing OP and advocates for the use of BRI as a valuable marker for early intervention approaches.

## Introduction

Osteoporosis (OP) is a widespread degenerative bone condition that impacts individuals globally. This condition is marked by a substantial reduction in bone mineral density (BMD) and deterioration of bone microstructure, resulting in greater bone fragility (1–3). Approximately 200 million people worldwide are affected by this disease, with a particularly high prevalence among women and the elderly. The incidence rate continues to rise (4). Due to frequent fractures and long-term rehabilitation needs, OP has become an urgent public health issue. By 2025, the direct medical costs related to this disease are expected to reach $25.3 billion annually, including costs for fracture treatment, rehabilitation, and indirect costs due to disability (5). This significantly increases the economic burden on individuals, families, and society, severely affecting patients' quality of life and social participation (6).

Obesity is a long-term metabolic disorder marked by excessive fat accumulation and metabolic alterations (7). It is not just an increase in body weight; more importantly, it is the abnormal accumulation of body fat, which leads to a range of health problems. Obesity has a profound impact on multiple chronic non-communicable diseases, including type 2 diabetes, cardiovascular conditions, and respiratory disorders (8). Additionally, obesity is closely associated with psychosocial disorders, such as depression (9). Precise measurement and evaluation of body fat are essential for comprehending the health effects of obesity. Historically, the Body Mass Index (BMI) has been the most commonly utilized indicator because of its ease of use and practicality. However, the limitations of BMI have gradually become apparent; BMI cannot distinguish between muscle and fat mass and is easily influenced by confounding factors (10).

Moreover, the relationship between obesity and OP shows a classic “obesity paradox,” where obesity is found to be a protective factor against OP in certain situations (11). This indicates that the complex relationship between obesity and bone metabolism requires further exploration.

Proposed by Thomas et al., the Body Roundness Index (BRI) is a new anthropometric measure designed to more precisely evaluate body fat distribution and related health risks. BRI calculation is based on an elliptical model of body shape, using eccentricity to estimate visceral and total body fat percentages (12). Unlike the traditional BMI, BRI considers not only weight and height but also incorporates waist circumference (WC), an important parameter. This allows BRI to more comprehensively reflect the distribution of visceral fat rather than just the total body fat. BRI has wide clinical applications. Studies have shown that BRI outperforms other traditional anthropometric measures in estimating the risk of various clinical endpoints such as kidney disease (13), cardiometabolic diseases (14, 15), and cancer (15). This means that BRI can provide more precise fat distribution assessments and more effectively predict and prevent various serious health issues related to obesity in clinical practice.

To date, no studies have established a relationship between BRI and total BMD. Using data from the National Health and Nutrition Examination Survey (NHANES), this research intends to examine the link between BRI and total BMD in U.S. adults aged 20 and above.

## Methods

### Data source and population study

NHANES, conducted by the National Center for Health Statistics (NCHS) under the Centers for Disease Control and Prevention (CDC), is a nationwide cross-sectional study aimed at comprehensively assessing the nutritional and health status of the U.S. population (16). This study provides scientific evidence for public health policy-making, disease prevention, and health promotion. The goal is to help public health policymakers, healthcare providers, and researchers better understand and address various health issues, thereby promoting the health and wellbeing of the entire population. The NCHS Ethics Review Board formally approved the study, and all participants gave written informed consent. For participants younger than 16, their legal guardians provided consent.

**Figure 1:**
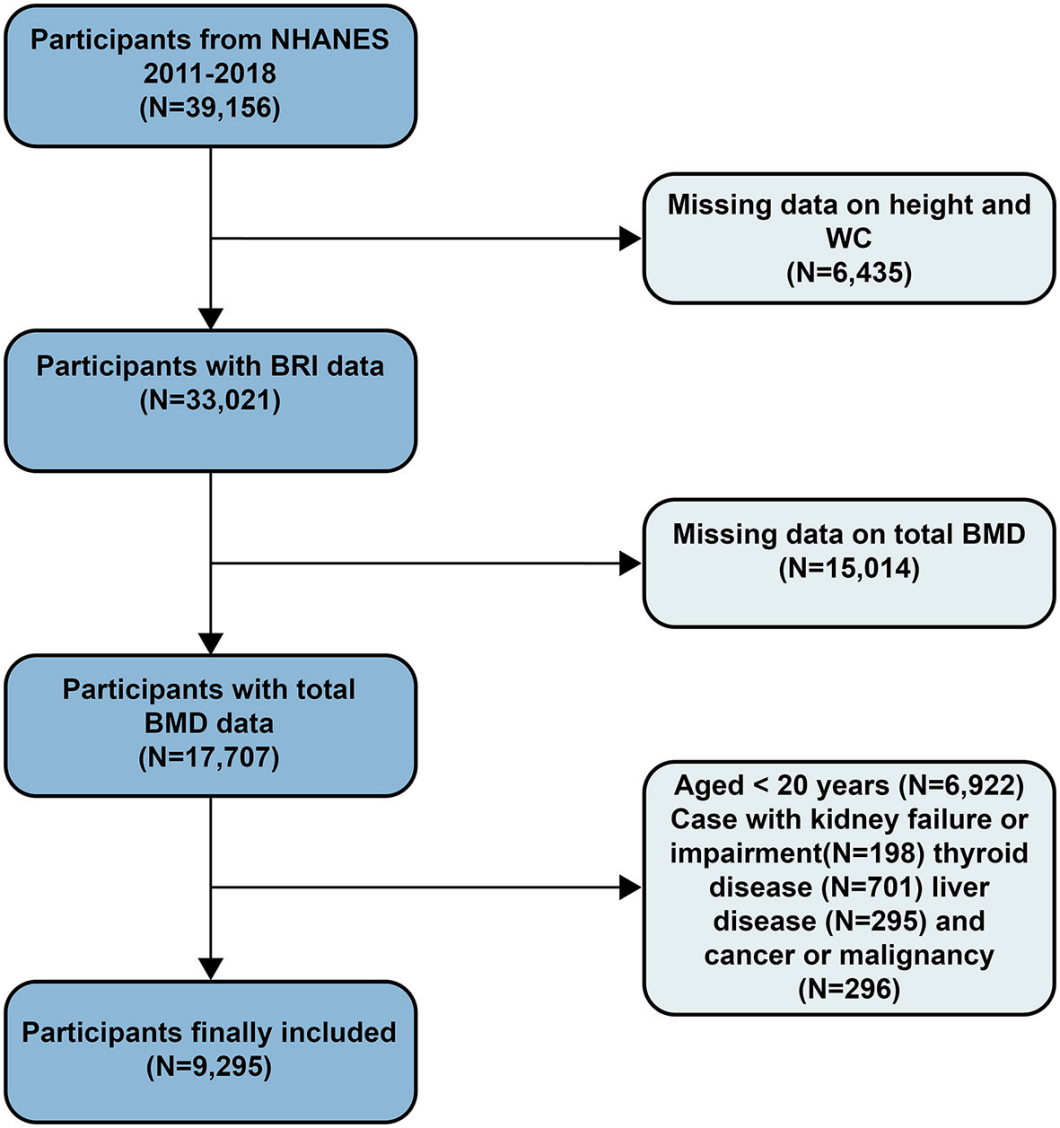
Study flowchart.

The data for this study covers the survey results from 2011 to 2018, with a total sample size of 39,156 individuals. The data includes health indicators, dietary records, and laboratory test results, ensuring comprehensiveness and reliability. Exclusion criteria included participants missing height and WC data (*N* = 6,435), those without total bone mineral density (BMD) data (*N* = 15,014), participants under 20 years old (*N* = 6,879), and those with kidney failure or impairment (*N* = 198), thyroid disease (*N* = 701), liver disease (*N* = 295), or cancer or malignancy (*N* = 296). After screening, the final sample size included in the study was 9,295 participants (Figure 1).

### BMD examination

In this study, BMD was measured using advanced dual-energy X-ray absorptiometry (DXA), a precise and widely utilized method for assessing bone mineral density (17). The equipment used was Hologic Discovery model A densitometers (Hologic, Inc., Bedford, Massachusetts), renowned for their high reliability and accuracy. All BMD measurements were performed by NHANES-certified radiologic technologists, ensuring the accuracy and consistency of the results.

### Definition of BRI

BRI is defined as follows: BRI = 364.2 – 365.5 × √[1 – (WC/(2π))^2^/(0.5 × height)^2^] (12). At mobile examination centers, measurements of height and WC were taken. This standardized measurement method ensures data consistency and accuracy.

### Covariate selection

In this study, we evaluated various covariates, including demographic characteristics, physical examination data, laboratory test results, and questionnaire data. The demographic data encompassed age, gender, race (including Mexican American, non-Hispanic White, non-Hispanic Black, and other races), education level (ranging from less than high school to college or higher), and the poverty-income ratio (PIR). Physical examination data included BMI, WC, and height. The laboratory data encompassed fasting plasma glucose (FPG), triglycerides (TG), total cholesterol (TC), and high-density lipoprotein cholesterol (HDL-C); measurements of vitamin D3 (25-OHD3), phosphorus, and total calcium; as well as levels of creatinine, alanine aminotransferase (ALT), and aspartate aminotransferase (AST). The questionnaire data covered diabetes status (self-reported diabetes, current use of insulin or other glucose-lowering medications, or an HbA1c level of 6.5% or higher), smoking status (having smoked at least 100 cigarettes in a lifetime), and alcohol consumption history (having consumed at least 4 drinks per day for women or 5 drinks per day for men). Additionally, it included information on physical activity (daily engagement in moderate exercise) and arthritis status. Comprehensive analysis of these covariates helps to deeply understand and interpret the study results.

### Data analysis

In this study, we performed detailed descriptive statistical analyses of the data. Survey-weighted linear regression was employed to evaluate the relationships between continuous variables, while survey-weighted chi-square tests were used to analyze differences in categorical variables. Means with 95% confidence intervals were used to present continuous variables, whereas percentages with 95% confidence intervals were used for categorical variables. We particularly focused on differences in baseline characteristics among participants grouped by BRI tertiles. To investigate whether BRI is independently associated with total BMD, we established three models. Model 1 is a weighted univariate linear regression model. Models 2 and 3 are weighted multivariate linear regression models. Model 2 was adjusted for gender, age, and race. Model 3 further included adjustments for education level, household income, BMI, HDL-C, TC, FPG, TG, 25-OHD3, phosphorus, total calcium, creatinine, ALT, AST, diabetes status, arthritis status, moderate physical activity, and smoking and drinking status. These adjustment variables were considered potential confounding factors. To explore non-linear relationships, we used smooth curve fitting and threshold effect analysis, particularly in analyzing the relationship between BRI and total BMD, using a recursive algorithm to identify inflection points and applying a two-segment linear regression model on either side of the inflection point. Subgroup analyses and log-likelihood ratio tests were used to evaluate interactions between subgroups. For missing data, we imputed continuous variables using medians or means based on the data distribution and categorical variables using modes. Analyses were performed using R software and Empower software, with statistical significance defined as a *p*-value below 0.05.

## Results

### Participant characteristics

The study included 9,295 participants, who were categorized into three groups according to their BRI values: low (1.05–3.89), medium (3.89–5.63), and high (5.63–19.10) (Table 1). Analysis showed significant differences among the BRI groups in terms of age, gender, race, diabetes status, arthritis condition, moderate physical activity, and a range of physical and laboratory indicators such as BMI, height, WC, FPG, TG, TC, HDL-C, total calcium, phosphorus, creatinine, ALT, and AST (*P* < 0.05). Compared to the lowest BRI group, the highest BRI group had a higher proportion of females and non-Hispanic Blacks and a higher proportion of participants with less than a high school education. Additionally, the highest BRI group had higher proportions of diabetes and arthritis patients, these participants typically did not engage in moderate daily physical activity and were older. In terms of physical and laboratory data, the highest BRI group had generally higher levels of BMI, WC, FPG, TG, TC, ALT, and AST, and lower levels of height, HDL-C, 25OHD3, phosphorus, total calcium, and creatinine. These differences reveal potential links between BRI and various health indicators, providing important baseline information for further research.

**Table 1:** Basic characteristics of the study population based on BRI tertiles.

**Variables**	**Low (*n* = 3,098)**	**Medium (*n* = 3,098)**	**High (*n* = 3,099)**	***P*-value**	***P*^†^-value**	***P^§^*-value**	***P^※^*-value**
	**(1.05, 3.89)**	**(3.89, 5.63)**	**(5.63, 19.10)**				
Age (years)	34.03 (33.34, 34.72)	40.51 (39.90, 41.12)	40.73 (40.15, 41.31)	< 0.0001	< 0.0001	0.5826	< 0.0001
Gender (%)				< 0.0001	0.0049	< 0.0001	0.0001
Male	53.97 (51.79, 56.13)	59.23 (56.46, 61.93)	47.19 (45.20, 49.18)				
Female	46.03 (43.87, 48.21)	40.77 (38.07, 43.54)	52.81 (50.82, 54.80)				
Race/ethnicity (%)				< 0.0001	< 0.0001	< 0.0001	< 0.0001
Mexican American	5.58 (4.33, 7.16)	12.04 (9.71, 14.83)	15.63 (12.61, 19.21)				
Non- Hispanic White	63.45 (59.50, 67.23)	59.66 (55.42, 63.76)	55.10 (50.44, 59.67)				
Non- Hispanic Black	12.07 (10.15, 14.29)	9.67 (7.97, 11.69)	14.38 (11.92, 17.23)				
Other race/ ethnicity	18.90 (16.56, 21.49)	18.63 (16.45, 21.02)	14.90 (13.21, 16.75)				
Education level (%)				< 0.0001	< 0.0001	0.0001	< 0.0001
Less than high school	9.80 (8.37, 11.44)	13.66 (11.88, 15.65)	15.91 (13.79, 18.28)				
High school or equivalent	18.97 (16.43, 21.79)	21.77 (19.43, 24.30)	25.58 (23.38, 27.92)				
College or above	71.23 (67.79, 74.45)	64.57 (61.21, 67.80)	58.51 (55.32, 61.63)				
Diabetes (%)				< 0.0001	< 0.0001	< 0.0001	< 0.0001
Yes	1.43 (1.01, 2.01)	4.65 (3.89, 5.56)	13.88 (12.44, 15.45)				
No	98.57 (97.99, 98.99)	95.35 (94.44, 96.11)	86.12 (84.55, 87.56)				
Arthritis (%)				< 0.0001	< 0.0001	0.0191	< 0.0001
Yes	6.32 (5.09, 7.81)	13.12 (11.44, 15.02)	16.29 (14.38, 18.40)				
No	93.68 (92.19, 94.91)	86.88 (84.98, 88.56)	83.71 (81.60, 85.62)				
Smoking (%)				0.0877	0.1257	0.4831	0.0284
Yes	37.35 (34.41, 40.37)	40.01 (37.32, 42.76)	41.42 (38.97, 43.92)				
No	62.65 (59.63, 65.59)	59.99 (57.24, 62.68)	58.58 (56.08, 61.03)				
Drinking (%)				0.0893	0.0446	0.9204	0.0830
Yes	11.04 (9.39, 12.95)	12.97 (11.52, 14.57)	12.87 (11.21, 14.74)				
No	88.96 (87.05, 90.61)	87.03 (85.43, 88.48)	87.13 (85.26, 88.79)				
Moderate activity (%)				< 0.0001	0.0058	< 0.0001	< 0.0001
Yes	54.81 (52.26, 57.34)	49.77 (47.20, 52.34)	42.21 (39.92, 44.54)				
No	45.19 (42.66, 47.74)	50.23 (47.66, 52.80)	57.79 (55.46, 60.08)				
Family PIR	2.98 (2.84, 3.11)	3.02 (2.91, 3.14)	2.70 (2.60, 2.80)	< 0.0001	0.4572	< 0.0001	< 0.0001
BMI (kg/cm^2^)	22.73 (22.60, 22.85)	27.82 (27.70, 27.95)	35.78 (35.49, 36.07)	< 0.0001	< 0.0001	< 0.0001	< 0.0001
Height (cm)	170.75 (170.36, 171.13)	169.94 (169.46, 170.42)	167.23 (166.78, 167.69)	< 0.0001	0.0091	< 0.0001	< 0.0001
WC (cm)	81.63 (81.25, 82.00)	96.31 (95.99, 96.63)	114.58 (113.95, 115.21)	< 0.0001	< 0.0001	< 0.0001	< 0.0001
FPG (mg/dL)	89.15 (88.31, 90.00)	95.30 (94.21, 96.38)	104.86 (103.19, 106.54)	< 0.0001	< 0.0001	< 0.0001	< 0.0001
TG (mg/dL)	107.85 (104.22, 111.47)	160.48 (153.58, 167.37)	176.79 (169.91, 183.68)	< 0.0001	< 0.0001	0.0005	< 0.0001
Total cholesterol (mg/dL)	180.20 (178.51, 181.89)	196.63 (194.20, 199.06)	193.88 (191.91, 195.85)	< 0.0001	< 0.0001	0.0668	< 0.0001
HDL-C (mg/dL)	59.06 (58.19, 59.94)	51.49 (50.66, 52.33)	47.12 (46.50, 47.75)	< 0.0001	< 0.0001	< 0.0001	< 0.0001
25OHD3 (nmol/L)	67.10 (65.22, 68.98)	63.84 (62.05, 65.64)	56.15 (54.46, 57.84)	< 0.0001	0.0012	< 0.0001	< 0.0001
Total calcium (mg/dL)	9.42 (9.40, 9.44)	9.38 (9.36, 9.40)	9.32 (9.30, 9.33)	< 0.0001	0.0002	< 0.0001	< 0.0001
Phosphorus (mg/dL)	3.79 (3.77, 3.81)	3.69 (3.66, 3.71)	3.67 (3.64, 3.70)	< 0.0001	< 0.0001	0.2789	< 0.0001
Creatinine (mg/dL)	0.86 (0.85, 0.87)	0.87 (0.86, 0.88)	0.82 (0.81, 0.83)	< 0.0001	0.0883	< 0.0001	< 0.0001
ALT (U/L)	21.53 (20.74, 22.32)	26.78 (25.89, 27.67)	29.57 (28.88, 30.26)	< 0.0001	< 0.0001	< 0.0001	< 0.0001
AST (U/L)	24.17 (23.52, 24.83)	25.08 (24.32, 25.85)	25.47 (24.79, 26.15)	0.0440	0.0942	0.4834	0.0155
Total BMD (g/cm^2^)	1.12 (1.11, 1.12)	1.12 (1.11, 1.13)	1.12 (1.11, 1.13)	0.5748	0.4195	0.9822	0.3544

Presented using mean values and their 95% confidence intervals for continuous variables, and percentage frequencies and their 95% confidence intervals for categorical variables.

^†^*P*-value for comparison between Low and Medium groups.

^§^*P*-value for comparison between Medium and High groups.

^※^*P*-value for comparison between Low and High groups. For pairwise comparisons, *p* < 0.05/3 is considered significant.

Family PIR, family poverty income ratio; BMI, body mass index; WC, waist circumference; FPG, fasting plasma glucose; TG, triglycerides; HDL-C, high-density lipoprotein cholesterol; ALT, alanine aminotransferase; AST, aspartate aminotransferase.

### Weighted multivariable linear regression analysis

Weighted multivariable linear regression models were utilized in this study to investigate the relationship between BRI and total BMD (Table 2). In the unadjusted model (Model 1), no significant positive correlation was found between BRI and total BMD (*P* = 0.4031). Furthermore, in a fully adjusted model (Model 3), after adjusting for potential confounders, each one-unit increase in BRI was associated with an average decrease of 0.0313 g/cm^2^ in total BMD (β = −0.0313, 95% CI: −0.0352, −0.0274), showing statistical significance (*P* < 0.0001). Additionally, by comparing groups divided by BRI tertiles, we found that in Model 3, the highest tertile group's total BMD was significantly lower by 0.0374 g/cm^2^ compared to the lowest tertile group (β = −0.0374, 95% CI: −0.0501, −0.0248), also showing high statistical significance (*P* < 0.0001) with a significant trend test (*P* for trend < 0.0001). The results indicate a significant negative association between BRI and total BMD after adjusting for covariates.

**Table 2:** Association between BRI and total BMD.

**Exposure**	**Model 1 β (95% CI), *P*-value**	**Model 2 β (95% CI), *P*-value**	**Model 3 β (95% CI), *P*-value**
BRI (continuous)	0.0005 (−0.0007, 0.0017), 0.4031	0.0027 (0.0015, 0.0039), < 0.0001	−0.0313 (−0.0352, −0.0274), < 0.0001
**BRI (quartile)**			
Low	Reference	Reference	Reference
Medium	0.0032 (−0.0045, 0.0109), 0.4195	0.0074 (0.0008, 0.0140), 0.0330	−0.0121 (−0.0195, −0.0046), 0.0032
High	0.0033 (−0.0036, 0.0102), 0.3544	0.0132 (0.0060, 0.0204), 0.0007	−0.0374 (−0.0501, −0.0248), < 0.0001
*P* for trend	0.3478	0.0008	< 0.0001

Model 1 was unadjusted; Model 2 was adjusted for sex, age, and ethnicity; and Model 3 further adjusted for education level, family PIR, BMI, HDL-C, TC, FPG, TG, 25-OHD3, phosphorus, total calcium, creatinine, ALT, AST, diabetes status, arthritis status, moderate activity status, drinking and smoking status.

### Smooth curve fitting to explore the correlation between BRI and total BMD

**Figure 2:**
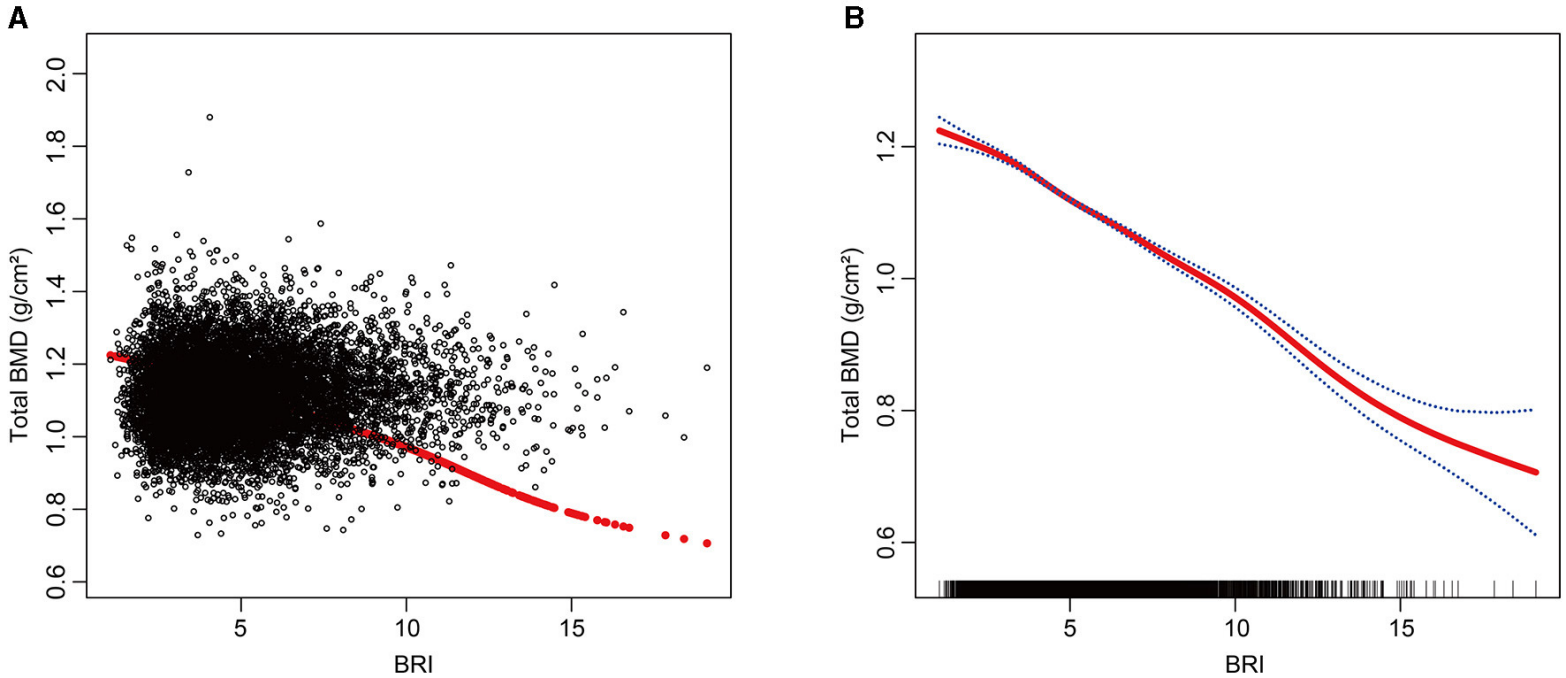
The Relationship between BRI and total BMD. **(A)** Each black dot on the graph denotes a single sample and the red line represents the fitted line for all participants. **(B)** The figure shows a smooth curve fit between the variables indicated by the red line. The blue line shows the 95% confidence interval of the fit.

To gain a deeper understanding of the relationship between BRI and total BMD in this study, we employed smooth curve fitting techniques, with a particular focus on the Generalized Additive Model (GAM). The analysis revealed a negative correlation between BRI and total BMD (Figure 2). To further examine this relationship, a threshold effect analysis was performed using a weighted two-segment linear regression model and a recursive algorithm. This analysis pinpointed an inflection point for BRI at 9.5229, with a likelihood ratio test *P*-value of 0.0050, indicating statistical significance. Below the BRI threshold of 9.5229, each one-unit increase in BRI corresponded to a 0.0298 g/cm^2^ decrease in total BMD (β = −0.0298, 95% CI: −0.0327, −0.0269). Above this threshold, each one-unit increase in BRI was linked to a larger decrease in total BMD of 0.0363 g/cm^2^ (β = −0.0363, 95% CI: −0.0409, −0.0318) (Table 3).

**Table 3:** Threshold effect analysis of BRI and total BMD.

**Total BMD**	**β (95% CI)**	***P*-value**
**BRI**		
Model I	−0.0313 (−0.0352, −0.0274)	< 0.0001
Model II		
Inflection point (K)	9.5229	
< K point effect 1	−0.0298 (−0.0327, −0.0269)	< 0.0001
>K point effect 2	−0.0363 (−0.0409, −0.0318)	< 0.0001
Effect 2 minus effect1	−0.0065 (−0.0111, −0.0020)	0.0049
Predicted value of the equation at the folding point	1.1227 (1.1168, 1.1286)	
Log-likelihood ratio test		0.0050

Adjusted for age, gender, ethnicity, education level, family PIR, BMI, HDL-C, TC, FPG, TG, 25-OHD3, phosphorus, total calcium, creatinine, ALT, AST, diabetes status, arthritis status, moderate activity status, drinking and smoking status. Model I: univariate linear regression; Model II: two-part regression model.

### Subgroup analysis

**Figure 3:**
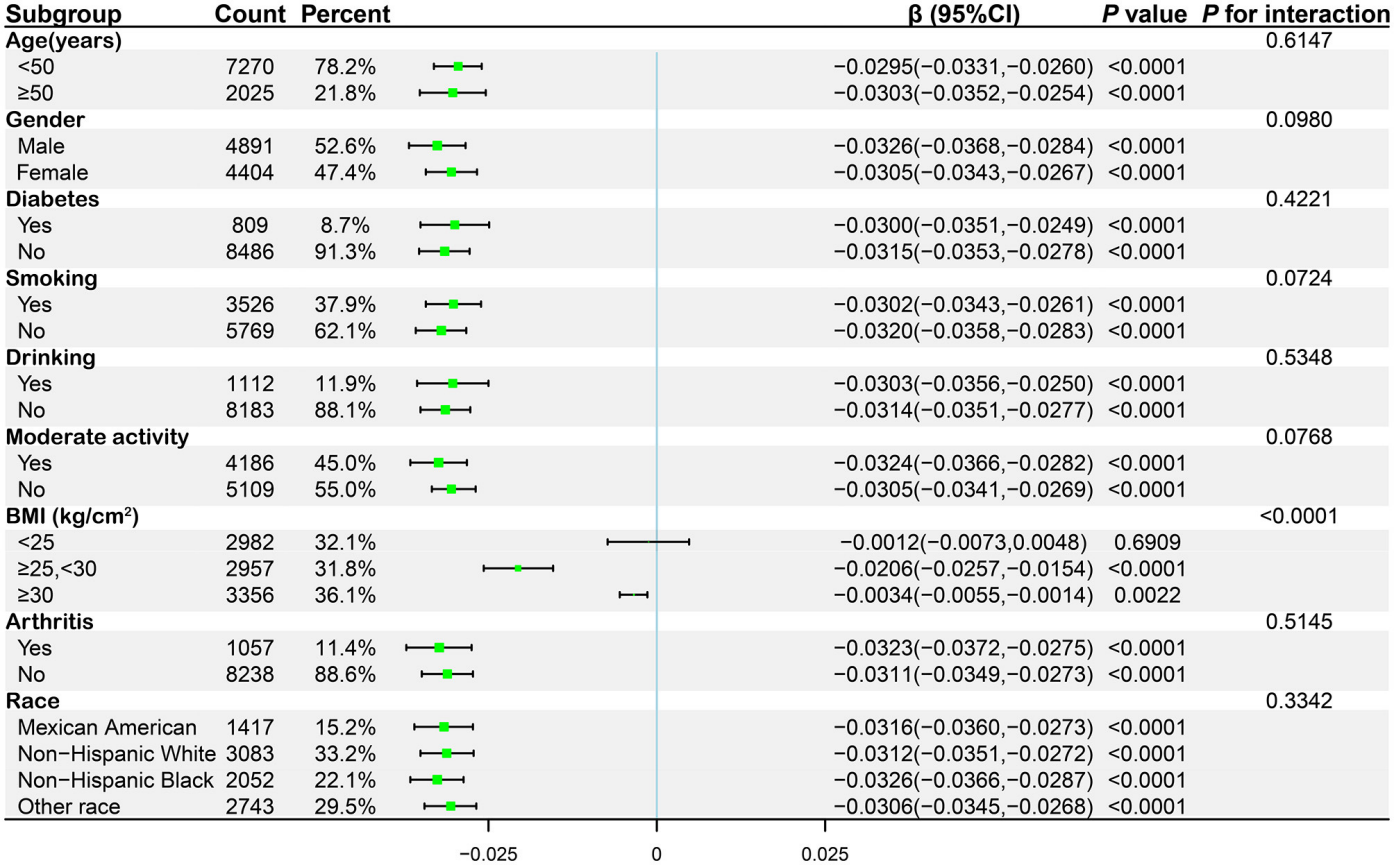
Subgroup analysis of the associations between BRI and total BMD.

**Figure 4:**
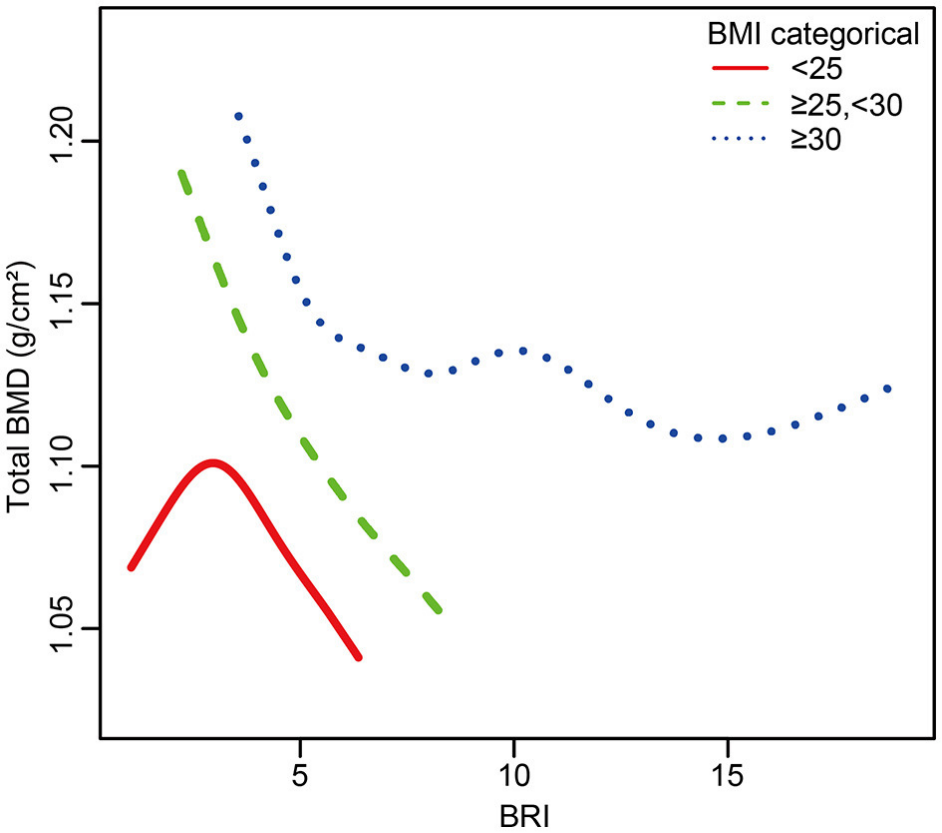
The relationship between BRI and total BMD grouped by BMI. The subgroup analyses were performed without adjusting for BMI.

This study utilized subgroup analyses and interaction tests to explore the association between BRI and total BMD across different populations. Participants were categorized based on various factors, including age, gender, history of diabetes and arthritis, smoking status, alcohol consumption, engagement in moderate physical activity, BMI, and race. In fully adjusted statistical models, BMI groups showed significant interaction effects (*P* for interaction < 0.05), suggesting that BMI may affect the relationship between BRI and total BMD differently (Figure 3). This negative correlation persisted across all relevant subgroups, and in all groups except for the BMI < 25 kg/m^2^ subgroup, *P-*values were < 0.05, demonstrating good intergroup stability. We further conducted smooth curve fitting based on BMI groups, revealing a complex inverted *U*-shaped association between BRI and BMD in the BMI < 25 kg/m^2^ group (Figure 4).

### Sensitivity analysis

To investigate the stability of the negative correlation between BRI and total BMD, we conducted a sensitivity analysis. Based on the BMD examination sites provided by NHANES, we selected the lumbar spine and pelvis for weighted linear regression analysis. The results showed that after considering the influence of measurement sites, the negative correlation between BRI and BMD remained significant, with β (95% CI) values of −0.0500 (−0.0571, −0.0430) and −0.0398 (−0.0464, −0.0332) respectively (see Supplementary Tables S1, S2).

Additionally, considering that menopausal status in women could be a potential confounding factor affecting the correlation between BRI and total BMD, we conducted a stratified analysis among the female population based on premenopausal and postmenopausal status and tested for interaction. The results showed that in the fully adjusted model, the negative correlation between BRI and total BMD remained significant in both premenopausal and postmenopausal women, with β (95% CI) values of −0.0163 (−0.0206, −0.0119) and −0.0161 (−0.0209, −0.0113) respectively, and the interaction test showed no significance in this stratification (see Supplementary Table S3). This indicates that the negative correlation between BRI and total BMD is stable and significant regardless of different BMD measurement sites or different menopausal statuses.

## Discussion

As far as we know, this is the first large-scale cross-sectional study using the NHANES database to investigate the correlation between BRI and total BMD. Our findings demonstrated a significant negative correlation between BRI and total BMD, consistent across various populations, suggesting a potential link between obesity and OP.

The relationship between obesity and BMD remains controversial. Some research indicates that obesity might have a protective impact on bone health, while a lower BMI is linked to reduced BMD and a higher risk of OP (18, 19). For example, Evans et al.'s study on individuals aged 55–75 found that obese participants had higher BMD compared to those with a normal BMI. The findings suggest that obesity may play a role in preventing age-related bone loss (20). Zhang et al.'s research, using data from the 2011–2018 NHANES, demonstrated a notable positive relationship between BMI and lumbar BMD, with each 1 kg/m^2^ increase in BMI associated with a 0.001 g/cm^2^ increase in lumbar BMD. This association remained significant and stable across all models (21).

However, contrasting findings exist. Jiao et al.'s study, based on NHANES data from 1999 to 2018 analyzing 11,615 adults, found an inverted *U*-shaped curve and negative correlation between different fat distributions (total fat, visceral fat, abdominal fat, and hip fat) and BMD (22). Wang et al.'s study based on 2011–2018 NHANES data found a significant negative correlation between the weight-adjusted waist index (WWI) and total BMD among U.S. adolescents. This relationship was consistent across all subgroups (except age) (23). These studies indicate that the relationship between obesity and BMD is complex and variable. Different populations and different obesity assessment criteria may affect the association between obesity and BMD, leading to entirely different conclusions. Therefore, to explore the complex relationship between obesity and OP, and given the excellent performance of BRI in assessing obesity in other contexts, we used NHANES data from 2011 to 2018 to explore the association between BRI and total BMD in adults aged 20 and older.

In our research, after adjusting for relevant covariates, a significant negative correlation between BRI and total BMD was observed. In fully adjusted statistical models, we found that BMI-grouped subgroups showed significant interaction effects (*P* for interaction < 0.05). This negative correlation persisted in all subgroups except for those with BMI < 25 kg/m^2^. Additionally, smooth curve fitting showed a negative correlation between BRI and total BMD. By applying a weighted two-segment linear regression model and a recursive algorithm, we identified an inflection point at BRI = 9.5229. Below this point, each one-unit increase in BRI corresponded to a decrease of 0.0298 g/cm^2^ in total BMD (β = −0.0298, 95% CI: −0.0327, −0.0269). Conversely, above the inflection point, each one-unit increase in BRI was linked to a larger decrease in total BMD of 0.0363 g/cm^2^ (β = −0.0363, 95% CI: −0.0409, −0.0318).

The inverse relationship between obesity and BMD can be attributed to several mechanisms. First, obesity increases the number of adipocytes in the bone marrow, altering their metabolic functions and prompting BMSCs to differentiate more into adipocytes rather than osteoblasts (24, 25). This shift leads to a rise in adipocytes and a reduction in osteoblasts within the bone marrow, causing an imbalance in bone cell activity and a decrease in bone turnover (26). Secondly, obesity is associated with increased levels of inflammatory cytokines. Inflammatory cytokines (such as IL-1, IL-6, and TNF-α) are released by adipose tissue, stimulating osteoclastogenesis and activation. These cytokines limit osteoprotegerin secretion, inhibit osteoblast differentiation, and accelerate the release of additional inflammatory and immune-regulating cytokines, thereby promoting osteoclast formation and increasing bone resorption (27–29). Additionally, leptin levels are higher in obese individuals. While leptin can inhibit osteoclastogenesis and promote the differentiation of stromal cells into osteoblasts *in vitro*, elevated levels of leptin are linked to decreased serotonin synthesis in hypothalamic neurons, impacting bone generation and overall damaging bone tissue (30–32). Adiponectin can promote the differentiation of BMSCs into osteoblasts, but its levels are lower in obese individuals, potentially negatively affecting BMD (33, 34). These mechanisms collectively contribute to the negative correlation between obesity and BMD.

We also analyzed potential reasons for the inflection point. This brings us to another classic theory about obesity: Frost's “mechanistic theory,” which suggests that bones adjust their mass and strength according to mechanical load (35). However, when fat tissue exceeds a certain amount (beyond the inflection point), this offsetting effect may diminish, and the negative impacts of obesity on bone metabolism may become more prominent. This combined effect may lead to the appearance of an inflection point. Additionally, in the smooth curve fitting by BMI groups, we observed a plateau around a BRI of 10 among individuals with a BMI higher than 30 kg/m^2^, which may further support our hypothesis. Subgroup analysis and smooth curve fitting by BMI groups suggest a more complex relationship between BRI-defined obesity and BMD, indicating the need for further research to uncover the underlying mechanisms.

In summary, our NHANES study uncovered a negative correlation between BRI and total BMD, enhancing our understanding of the intricate relationship between obesity and OP. This study boasts several strengths: it utilized a large, representative sample, minimized population heterogeneity, adjusted for numerous confounders, and included subgroup analyses to ensure the robustness and validity of the conclusions. Notably, this is the first NHANES study to explore the correlation between BRI and total BMD, highlighting its innovative approach. However, the study also has its limitations. As a cross-sectional study, it lacks a temporal dimension and cannot establish causal relationships between the variables. Additionally, total BMD may be influenced by various factors such as lifestyle, dietary habits, and environmental influences. Although we adjusted for well-known covariates related to BMD, it was not possible to account for all potential confounders.

## Conclusions

A significant negative correlation between BRI and total BMD was revealed in this study. This finding is crucial for understanding the relationship between obesity and OP, suggesting that BRI could serve as a potential indicator for OP prevention. Maintaining appropriate levels of BRI is essential. Such research will enhance our ability to implement precise and early interventions, ultimately improving the prognosis for populations at high risk of OP.

## Data availability statement

The original contributions presented in the study are included in the article/Supplementary material, further inquiries can be directed to the corresponding author.

## Ethics statement

The studies involving humans were approved by National Center for Health Statistics Ethics Review Committee. The studies were conducted in accordance with the local legislation and institutional requirements. The participants provided their written informed consent to participate in this study.

## Author contributions

ZD: Data curation, Methodology, Software, Visualization, Writing – original draft, Writing – review & editing. ZZ: Writing – original draft. RT: Conceptualization, Writing – original draft. XQ: Data curation, Writing – original draft. ZH: Project administration, Writing – original draft. MS: Writing – original draft. FY: Funding acquisition, Writing – original draft, Writing – review & editing.

## References

[1] Kanis JA McCloskey EV Johansson H Cooper C Rizzoli R Reginster J-Y. European guidance for the diagnosis and management of osteoporosis in postmenopausal women. Osteopor Int. (2013) 24:23–57. 10.1007/s00198-012-2074-y 23079689 PMC3587294

[2] Kanis JA Delmas P Burckhardt P Cooper C Torgerson Do. Guidelines for diagnosis and management of osteoporosis. Osteopor Int. (1997) 7:390–406. 10.1007/BF01623782 9373575

[3] Qaseem A Forciea MA McLean RM Denberg TD Clinical Guidelines Committee of the American College of Physicians. Treatment of low bone density or osteoporosis to prevent fractures in men and women: a clinical practice guideline update from the American College of Physicians. Ann Int Med. (2017) 166:818–39. 10.7326/M15-1361 28492856

[4] Kushchayeva Y Pestun I Kushchayev S Radzikhovska N Lewiecki EM. Advancement in the treatment of osteoporosis and the effects on bone healing. J Clin Med. (2022) 11:7477. 10.3390/jcm11247477 36556093 PMC9781093

[5] Dempster DW. Osteoporosis and the burden of osteoporosis-related fractures. Am J Manag Care. (2011) 17:S164. 21761955

[6] Khosla S Hofbauer LC. Osteoporosis treatment: recent developments and ongoing challenges. Lancet Diabetes Endocrinol. (2017) 5:898–907. 10.1016/S2213-8587(17)30188-2 28689769 PMC5798872

[7] Alberti KG Eckel RH Grundy SM Zimmet PZ Cleeman JI Donato KA. Harmonizing the metabolic syndrome: a joint interim statement of the international diabetes federation task force on epidemiology and prevention; national heart, lung, and blood institute; American heart association; world heart federation; international atherosclerosis society; and international association for the study of obesity. Circulation. (2009) 120:1640–5. 10.1161/CIRCULATIONAHA.109.192644 19805654

[8] Rao W-W Zong Q-Q Zhang J-W An F-R Jackson T Ungvari GS. Obesity increases the risk of depression in children and adolescents: Results from a systematic review and meta-analysis. J Affect Disord. (2020) 267:78–85. 10.1016/j.jad.2020.01.154 32063576

[9] Milaneschi Y Simmons WK van Rossum EF Penninx BW. Depression and obesity: evidence of shared biological mechanisms. Mol Psychiatry. (2019) 24:18–33. 10.1038/s41380-018-0017-5 29453413

[10] Khan I Chong M Le A Mohammadi-Shemirani P Morton R Brinza C. Surrogate adiposity markers and mortality. JAMA Netw Open. (2023) 6:e2334836–e2334836. 10.1001/jamanetworkopen.2023.34836 37728925 PMC10512100

[11] Torres-Costoso A Garrido-Miguel M Gracia-Marco L López-Muñoz P Reina-Gutiérrez S Núñez de Arenas-Arroyo S. The “Fat but Fit” paradigm and bone health in young adults: a cluster analysis. Nutrients. (2021) 13:518. 10.3390/nu13020518 33562503 PMC7914522

[12] Thomas DM Bredlau C Bosy-Westphal A Mueller M Shen W Gallagher D. Relationships between body roundness with body fat and visceral adipose tissue emerging from a new geometrical model. Obesity. (2013) 21:2264–71. 10.1002/oby.20408 23519954 PMC3692604

[13] Zhang Y Gao W Ren R Liu Y Li B Wang A. Body roundness index is related to the low estimated glomerular filtration rate in Chinese population: a cross-sectional study. Front Endocrinol. (2023) 14:1148662. 10.3389/fendo.2023.1148662 37056676 PMC10086436

[14] Cai X Song S Hu J Zhu Q Yang W Hong J. Body roundness index improves the predictive value of cardiovascular disease risk in hypertensive patients with obstructive sleep apnea: a cohort study. Clin Exp Hypertens. (2023) 45:2259132. 10.1080/10641963.2023.2259132 37805984

[15] Rico-Martín S Calderón-García JF Sánchez-Rey P Franco-Antonio C Martinez Alvarez M Sánchez Muñoz-Torrero JF. Effectiveness of body roundness index in predicting metabolic syndrome: a systematic review and meta-analysis. Obes Rev. (2020) 21:e13023. 10.1111/obr.13023 32267621

[16] Fain JA. NHANES: Use of a Free Public Data Set. Los Angeles, CA: SAGE Publications (2017). p. 151.

[17] Ouyang Y Quan Y Guo C Xie S Liu C Huang X. Saturation effect of body mass index on bone mineral density in adolescents of different ages: a population-based study. Front Endocrinol. (2022) 13:922903. 10.3389/fendo.2022.922903 35865310 PMC9294630

[18] Asomaning K Bertone-Johnson ER Nasca PC Hooven F Pekow PS. The association between body mass index and osteoporosis in patients referred for a bone mineral density examination. J Womens Health. (2006) 15:1028–34. 10.1089/jwh.2006.15.1028 17125421

[19] Ravn P Cizza G Bjarnason N Thompson D Daley M Wasnich R. Low body mass index is an important risk factor for low bone mass and increased bone loss in early postmenopausal women. J Bone Miner Res. (1999) 14:1622–7. 10.1359/jbmr.1999.14.9.1622 10469292

[20] Evans AL Paggiosi MA Eastell R Walsh JS. Bone density, microstructure and strength in obese and normal weight men and women in younger and older adulthood. J Bone Miner Res. (2015) 30:920–8. 10.1002/jbmr.2407 25400253

[21] Zhang Y Tan C Tan W. BMI socioeconomic status, and bone mineral density in US adults: mediation analysis in the NHANES. Front Nutr. (2023) 10:1132234. 10.3389/fnut.2023.1132234 36960203 PMC10027781

[22] Jiao Y Sun J Li Y Zhao J Shen J. Association between adiposity and bone mineral density in adults: insights from a national survey analysis. Nutrients. (2023) 15:3492. 10.3390/nu15153492 37571429 PMC10420642

[23] Wang X Yang S He G Xie L. The association between weight-adjusted-waist index and total bone mineral density in adolescents: NHANES 2011–2018. Front Endocrinol. (2023) 14:1191501. 10.3389/fendo.2023.1191501 37265707 PMC10231032

[24] Zong Q Bundkirchen K Neunaber C Noack S. Are the properties of bone marrow-derived mesenchymal stem cells influenced by overweight and obesity? Int J Mol Sci. (2023) 24:4831. 10.3390/ijms24054831 36902259 PMC10003331

[25] Khan AU Qu R Fan T Ouyang J Dai J. A glance on the role of actin in osteogenic and adipogenic differentiation of mesenchymal stem cells. Stem Cell Res Ther. (2020) 11:283. 10.1186/s13287-020-01789-2 32678016 PMC7364498

[26] Fintini D Cianfarani S Cofini M Andreoletti A Ubertini G Cappa M. The bones of children with obesity. Front Endocrinol. (2020) 11:200. 10.3389/fendo.2020.00200 32390939 PMC7193990

[27] Wei S Kitaura H Zhou P Ross FP Teitelbaum SL. IL-1 mediates TNF-induced osteoclastogenesis. J Clin Invest. (2005) 115:282–90. 10.1172/JCI200523394 15668736 PMC544608

[28] Wedell-Neergaard A-S Lehrskov LL Christensen RH Legaard GE Dorph E Larsen MK. Exercise-induced changes in visceral adipose tissue mass are regulated by IL-6 signaling: a randomized controlled trial. Cell Metab. (2019) 29:844–55. e3. 10.1016/j.cmet.2018.12.007 30595477

[29] Zhang K Wang C Chen Y Ji X Chen X Tian L. Preservation of high-fat diet-induced femoral trabecular bone loss through genetic target of TNF-α. Endocrine. (2015) 50:239–49. 10.1007/s12020-015-0554-5 25700562

[30] Karsenty G Ferron M. The contribution of bone to whole-organism physiology. Nature. (2012) 481:314–20. 10.1038/nature10763 22258610 PMC9047059

[31] Ruhl CE Everhart JE. Relationship of serum leptin concentration with bone mineral density in the United States population. J Bone Miner Res. (2002) 17:1896–903. 10.1359/jbmr.2002.17.10.1896 12369793

[32] Rinonapoli G Pace V Ruggiero C Ceccarini P Bisaccia M Meccariello L. Obesity and bone: a complex relationship. Int J Mol Sci. (2021) 22:13662. 10.3390/ijms222413662 34948466 PMC8706946

[33] Pu Y Wang M Hong Y Wu Y Tang Z. Adiponectin promotes human jaw bone marrow mesenchymal stem cell chemotaxis via CXCL 1 and CXCL 8. J Cell Mol Med. (2017) 21:1411–9. 10.1111/jcmm.13070 28176455 PMC5487911

[34] Yamauchi T Kamon J Waki H Terauchi Y Kubota N Hara K. The fat-derived hormone adiponectin reverses insulin resistance associated with both lipoatrophy and obesity. Nat Med. (2001) 7:941–6. 10.1038/90984 11479627

[35] Frost HM. Bone's mechanostat: a 2003 update. Anat Rec A Discov Mol Cell Evol Biol. (2003) 275:1081–101. 10.1002/ar.a.10119 14613308

