# Supplementary material for: Negative association between Body Roundness Index and bone mineral density: insights from NHANES
Source: Front Nutr. 2024 Aug 8;11:1448938. doi: 10.3389/fnut.2024.1448938 (PMC11340502; doi:10.3389/fnut.2024.1448938)
Supplement: Supplementary file 1 [file Table_1.DOCX]

**Table S1. Association between BRI and Lumbar BMD**

| **Exposure** | **Model 1 β (95% CI),**  ***P* value** | **Model 2 β (95% CI),**  ***P* value** | **Model 3 β (95% CI),**  ***P* value** |
| --- | --- | --- | --- |
| BRI (continuous) | -0.0062(-0.0080,-0.0043)  <0.0001 | -0.0055(-0.0075,-0.0034)  <0.0001 | -0.0500(-0.0571,-0.0430)  <0.0001 |
| BRI (quartile) |  |  |  |
| Low | Reference | Reference | Reference |
| Medium | -0.0253(-0.0361,-0.0146)  <0.0001 | -0.0150(-0.0258,-0.0043)  0.0084 | -0.0415(-0.0547,-0.0283)  <0.0001 |
| High | -0.0487(-0.0599,-0.0376)  <0.0001 | -0.0427(-0.0549,-0.0306)  <0.0001 | -0.1136(-0.1361,-0.0912)  <0.0001 |
| *P* for trend | <0.0001 | <0.0001 | <0.0001 |

Caption: Model 1 was unadjusted; Model 2 was adjusted for sex, age, and ethnicity; and Model 3 further adjusted for education level, family PIR, BMI, HDL-C, TC, FPG, TG, 25-OHD3, phosphorus, total calcium, creatinine, ALT, AST, diabetes status, arthritis status, moderate activity status, drinking and smoking status.

**Table S2. Association between BRI and Pelvis BMD**

| **Exposure** | **Model 1 β (95% CI),**  ***P* value** | **Model 2 β (95% CI),**  ***P* value** | **Model 3 β (95% CI),**  ***P* value** |
| --- | --- | --- | --- |
| BRI (continuous) | 0.0063(0.0038,0.0089)  <0.0001 | 0.0092(0.0066,0.0118)  <0.0001 | -0.0398(-0.0464,-0.0332)  <0.0001 |
| BRI (quartile) |  |  |  |
| Low | Reference | Reference | Reference |
| Medium | 0.0625(0.0513,0.0736)  <0.0001 | 0.0766(0.0668,0.0864)  <0.0001 | 0.0598(0.0477,0.0719)  <0.0001 |
| High | 0.0630(0.0510,0.0750)  <0.0001 | 0.0817(0.0695,0.0938)  <0.0001 | 0.0489(0.0255,0.0723)  0.0002 |
| *P* for trend | <0.0001 | <0.0001 | <0.0001 |

Caption: Model 1 was unadjusted; Model 2 was adjusted for sex, age, and ethnicity; and Model 3 further adjusted for education level, family PIR, BMI, HDL-C, TC, FPG, TG, 25-OHD3, phosphorus, total calcium, creatinine, ALT, AST, diabetes status, arthritis status, moderate activity status, drinking and smoking status.

**Table S3. Subgroup analysis of the associations between BRI and Total BMD**

| **Subgroup** | **Count** | **Percent** | **β (95% CI)** | ***P* value** | ***P* for interaction** |
| --- | --- | --- | --- | --- | --- |
| **menopausal status** |  |  |  |  | 0.9286 |
| premenopausal | 3327 | 75.6% | -0.0163(-0.0206,-0.0119) | <0.0001 |  |
| postmenopausal | 1076 | 24.4% | -0.0161(-0.0209,-0.0113) | <0.0001 |  |

Adjusted for age, and ethnicity, education level, family PIR, BMI, HDL-C, TC, FPG, TG, 25-OHD3, phosphorus, total calcium, creatinine, ALT, AST, diabetes status, arthritis status, moderate activity status, drinking and smoking status.
